# Stereological study of the neuronal number and volume of 38 brain subdivisions of subjects diagnosed with autism reveals significant alterations restricted to the striatum, amygdala and cerebellum

**DOI:** 10.1186/s40478-014-0141-7

**Published:** 2014-09-18

**Authors:** Jerzy Wegiel, Michael Flory, Izabela Kuchna, Krzysztof Nowicki, Shuang Yong Ma, Humi Imaki, Jarek Wegiel, Ira L Cohen, Eric London, Thomas Wisniewski, William Ted Brown

**Affiliations:** Department of Developmental Neurobiology, NYS Institute for Basic Research in Developmental Disabilities, 1050 Forest Hill Road, Staten Island, NY 10314 USA; Department of Infant Development, NYS Institute for Basic Research in Developmental Disabilities, 1050 Forest Hill Road, Staten Island, NY 10314 USA; Department of Psychology, NYS Institute for Basic Research in Developmental Disabilities, 1050 Forest Hill Road, Staten Island, NY 10314 USA; Department of Neurology, Psychiatry and Pathology, New York University School of Medicine, 450 East 29th Street, Room 802, New York, NY 10016 USA; Department of Human Genetics, NYS Institute for Basic Research in Developmental Disabilities, 1050 Forest Hill Road, Staten Island, NY 10314 USA

**Keywords:** Autism, Brain, Volume, Number of neurons, Cerebellum, Striatum, Amygdala

## Abstract

**Introduction:**

A total of 38 brain cytoarchitectonic subdivisions, representing subcortical and cortical structures, cerebellum, and brainstem, were examined in 4- to 60-year-old subjects diagnosed with autism and control subjects (a) to detect a global pattern of developmental abnormalities and (b) to establish whether the function of developmentally modified structures matches the behavioral alterations that are diagnostic for autism. The volume of cytoarchitectonic subdivisions, neuronal numerical density, and total number of neurons per region of interest were determined in 14 subjects with autism and 14 age-matched controls by using unbiased stereological methods.

**Results:**

The study revealed that significant differences between the group of subjects with autism and control groups are limited to a few brain regions, including the cerebellum and some striatum and amygdala subdivisions. In the group of individuals with autism, the total number and numerical density of Purkinje cells in the cerebellum were reduced by 25% and 24%, respectively. In the amygdala, significant reduction of neuronal density was limited to the lateral nucleus (by 12%). Another sign of the topographic selectivity of developmental alterations in the brain of individuals with autism was an increase in the volumes of the caudate nucleus and nucleus accumbens by 22% and 34%, respectively, and the reduced numerical density of neurons in the nucleus accumbens and putamen by 15% and 13%, respectively.

**Conclusions:**

The observed pattern of developmental alterations in the cerebellum, amygdala and striatum is consistent with the results of magnetic resonance imaging studies and their clinical correlations, and of some morphometric studies that indicate that detected abnormalities may contribute to the social and communication deficits, and repetitive and stereotypical behaviors observed in individuals with autism.

## Introduction

Autism is a lifelong disorder characterized by disrupted development of social and communication skills and restricted, repetitive, and stereotypical patterns of behavior, interests, and activities. In nearly 50% of individuals later diagnosed with autism, functional alterations are noted at between 14 and 24 months, and alterations are observed in all three diagnostic functional domains at 24 months of age [1,2].

In the past three decades, numerous studies have been conducted on the trajectory of brain overgrowth, differences between the sizes of individual brain structures and regions, and the abnormal number and size of neurons in the brain in autism spectrum disorder (ASD). A large cluster of studies has focused on distortion of the trajectory of brain growth, which may involve modifications of the brain and of brain structure volumes as well as of the number of neurons. A rapid increase in head circumference at 1–2 years of age [3-7] followed by a slower rate of brain growth between 2 and 4 years of age [8-11] reportedly results in only a 2% larger brain size in adult patients with autism [12] or a smaller brain size [13] in comparison to control subjects. However, recent analysis by Raznahan et al. [14] revealed that the vast majority (83%) of head circumference studies in ASD used head circumference norms as comparison data. Reassessment of evidence of early brain overgrowth revealed that studies using head circumference norms are significantly more likely to identify head circumference abnormalities in ASD than are comparisons between head circumference data of children with ASD and locally recruited control subjects [14]. The authors of this analysis suggest that reports of early brain overgrowth in children with ASD reflect replicable Centers for Disease Control and Prevention norm biases rather than a disease-related phenomenon. However, there is no doubt that in several genetic syndromes characterized by autistic-like behavior, the brain is enlarged [15], or affected individuals are microcephalic [16,17].

Individual brain structures have distinct developmental profiles from the trajectory of brain development [18-20]. Growth abnormalities of various brain regions involved in cognitive, social, and emotional functions and language development are considered to be a reflection of their contribution to the clinical autism phenotype [9-11,21]. Some postmortem studies suggest that volumetric changes are associated with an increase or decrease in the number of neurons. Courchesne et al. [22] revealed 67% more neurons in the prefrontal cortex in 2- to 16-year-old individuals with autism and linked these differences to prenatal disturbances of mechanisms that govern proliferation, cell cycle regulation, and apoptosis. Santos et al. [23] demonstrated insignificantly more von Economo neurons in the frontoinsular cortex in 4- to 11-year-old children diagnosed with autism than in controls (*p* = 0.054), but the contribution of this small population of neurons to the volume of this cortical region is marginal. However, the majority of reports show that the numbers of many neuronal populations in the brain structures of individuals with autism are unmodified [23-28] or regionally decreased [29-34].

This project was designed to partially reduce the limitations of research outcome due to the small size of the cohort examined with (a) different methods of tissue preservation and evaluation and (b) concentration of the study on a few brain regions. Four parameters—brain weight, the volume of brain structures’ cytoarchitectonic subdivisions, the total number of neurons, and the numerical density of neurons per region of interest—were selected to detect developmental and age-associated alterations in the brains of 14 individuals diagnosed with autism and 14 control subjects. The aim of the study of 14 brain structures and their 24 subdivisions (layers, sectors, nuclei) including subcortical and cortical structures, cerebellum, and brainstem was to identify a global pattern of alterations and to establish whether the function of altered structures matches the behavioral alterations that are diagnostic for autism.

## Materials and methods

Originally, 39 brains were assigned to this study of developmental abnormalities in autism, including 21 brains of subjects diagnosed with autism and 18 control brains. Application of clinical inclusion criteria and neuropathological exclusion criteria reduced the size of this cohort to 28 subjects, comprising 14 subjects with autism and 14 age-matched control individuals. Two cases did not meet the Autism Diagnostic Interview-Revised (ADI-R) [1] criteria for diagnosis of autism. One affected subject and four control subjects were excluded because of severe brain autolysis, and the brains of three subjects with autism were excluded because of hypoxic encephalopathy, and another one because of multiple microinfarcts [35].

The clinical diagnosis of autism was confirmed by a postmortem application of the ADI-R. Results of the ADI-R and the clinical characteristics of this cohort have been summarized by Wegiel et al. [35]. Eight subjects with autism (57%) were affected by mild to severe intellectual deficits, which were detected using the Wechsler Intelligence Scale for Children III and the Woodcock-Johnson Tests of Achievement-Revised. Seven of the 14 subjects with autism were diagnosed with seizures (50%), and in five cases (36%), death was seizure-related.

Neuropathological evaluation revealed various types of developmental abnormalities in 13/14 brains of individuals diagnosed with autism (93%), which were summarized in our previous report [36], including (a) defects of proliferation with subependymal nodular dysplasia, (b) defects of migration resulting in subcortical and periventricular heterotopias, and (c) dysplastic changes in the neocortex, archicortex, dentate gyrus, Ammons horn, and cerebellum.

The study has been approved by the institutional review board (Application # 463) of the New York State Institute for Basic Research in Developmental Disabilities.

### Tissue processing, embedding, sectioning, and staining

Tissue samples, demographics of individuals with autism and control subjects, brain weight, and changes during processing were characterized in our previous report [35]. In short, the postmortem interval (PMI), corresponding to the period between death and autopsy, ranged from 6 to 28 hours in the control group, and from 3 to 50 hours in the affected group, but the difference in PMI between the two groups was not significant. The average weight of the brains in the cohort with autism (1,453 g) was not significantly different from that in the control group (1,372 g). The brain hemisphere was fixed with 10% buffered formalin for an average of 408 days in the control group and 905 days in the affected group. Brain dehydration in a series of ascending concentrations of ethyl alcohol for 36 and 38 days in the control and affected groups, respectively, resulted in reduction of brain hemisphere weight by 47% on average in the group of subjects diagnosed with autism and by 45% in the control group. Brain hemispheres were embedded in 8% celloidin [37]. Serial 200-μm-thick sections stained with cresyl violet and mounted with Acrytol were used for morphometric studies.

### Delineation of regions of interest

**Figure 1:**
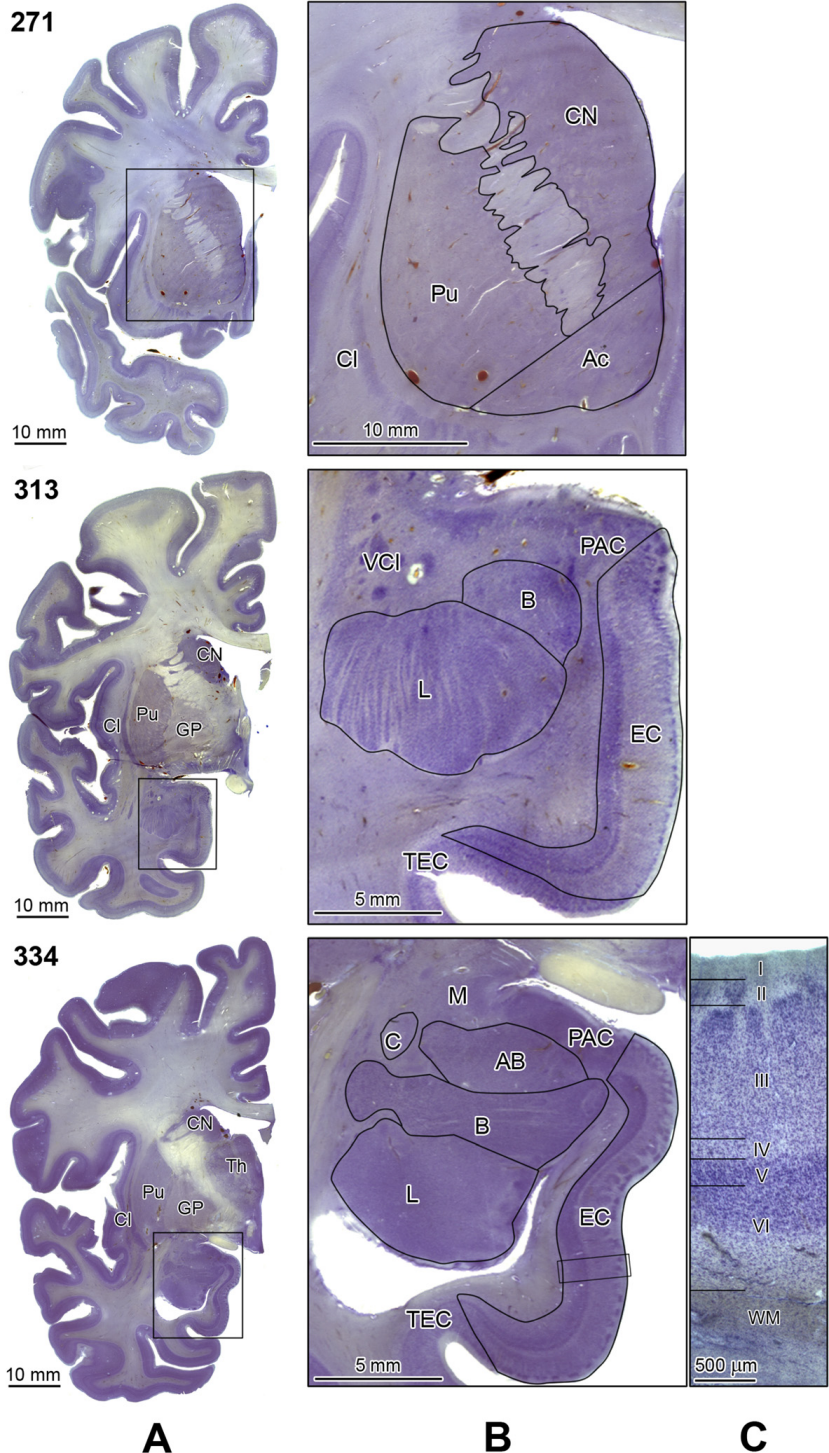
**Delineation of the striatum subdivisions, the amygdala nuclei and anterior portion of the entorhinal cortex.** Panel **A** shows three serial coronal sections (271, 313, and 334) stained with cresyl violet. Rectangles mark inserts that are shown enlarged in panel **B**. Section 271 shows delineation of the caudate nucleus (CN), putamen (Pu) and nucleus accumbens (Ac). Enlargements of sections 313 and 334 demonstrate the borders of the lateral (L), basal (B), accessory basal (AB), and central (C) nuclei within the amygdaloid complex. The adjacent median (M) nucleus, preamygdaloid cortex (PAC), and ventral claustrum (VCl) are marked. The borders of the enorhinal cortex (EC) are delinated including the borderline with the transentorhinal cortex (TEC) and PAC. Higher magnification (Panel **C**) shows EC layers typical for the mid-level of the rostrocaudal EC extension including layer I (molecular layer); islands of stellate neurons in layer II; a broad layer III; and acellular layer IV. On this level large darkly stained neurons form narrow layer (V), whereas the broad layer VI is built up of the smallest neurons, with decreasing gradient of neuronal density in the deeper part of this layer and a diffuse border with white matter (WM). Other examined structures: globus pallidus (GP), claustrum (Cl), and thalamus (Th). Calibration bar length: 10 mm, 5 mm, and 500 μm.

**Figure 2:**
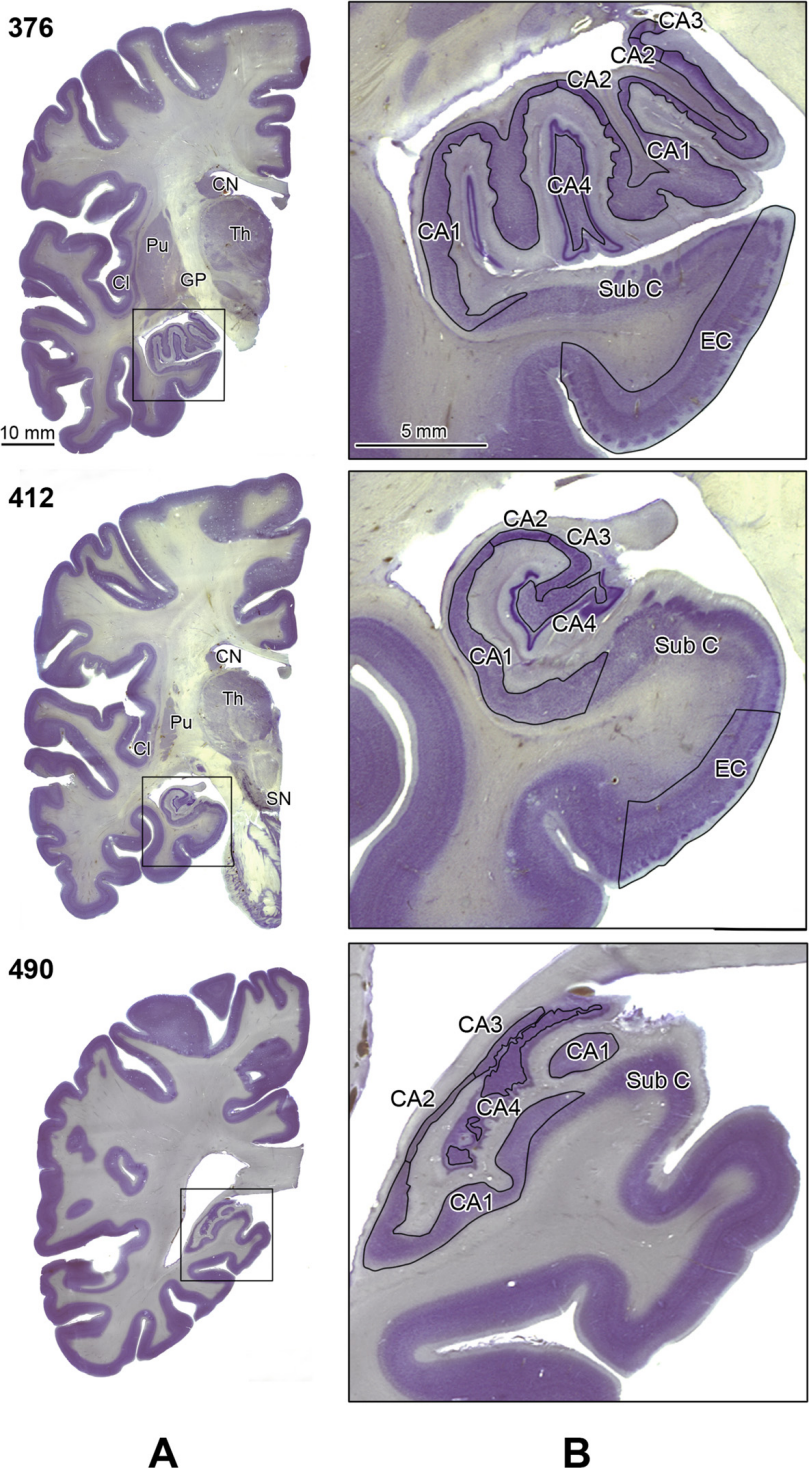
**Delination of the Ammons horn sectors and the posterior portion of the entorhinal cortex.** Panel A shows CV-stained hemispheric sections on the level of the hippocampal head (s. 376), body (s. 412) and tail (s. 490). The rectangle in panel **A** marks an area that was enlarged 5-fold and is shown in panel **B**. Stereologically examined structures are labeled as: sectors CA1, 2, 3, 4; EC, entorhinal cortex; CN, caudate nucleus; Pu, putamen; GP, globus pallidus; Cl, claustrum; Th, thalamus; and SN, substantia nigra. The subicular complex is labeled as Sub C. Calibration bar length: 10 mm and 5 mm.

The anatomical boundaries of the examined brain structures and their cytoarchitectonic subdivisions were described in detail in our previous report [35]. Delineation of structures requiring micrographs is illustrated with Figures 1 and 2, which show partitioning of the striatum (caudate nucleus, putamen, nucleus accumbens and globus pallidus); the amygdala (lateral, basal, accessory basal and central nuclei); layers of the entorhinal cortex; and sectors in the Ammons horn.

The rostral striatum is divided into the caudate nucleus and the putamen by the internal capsule. The border between the nucleus accumbens, the caudate nucleus and the putamen was arbitrarily defined by a line perpendicular to the midline of the internal capsule at its ventral end [38]. The number of striatal small neurons was estimated but a few dispersed large neurons [39] were not included in this study. The external medullary lamina marks the border between the putamen and the globus pallidus. The medial medullary lamina divides the globus pallidus into the external and internal pallidum [40]. The number of neurons was determined in both parts of the pallidum. The boundaries of the lateral, basal, accessory basal and central nuclei were delinated using cytoarchitectonic criteria described in detail by Schumann and Amaral [31,41]. The entorhinal cortex (EC, Brodmann area 28) occupies the gyrus ambiens and the anterior portion of the hippocampal gyrus. The anterior border of the EC starts a few millimeters rostrally to the amygdala complex. The EC ends rostrally to the lateral geniculate body. The medial portion of the EC borders with the preamygdaloid cortex and caudally with the subicular complex. The lateral portion of the EC borders with Brodmann area 35, which lacks a distinct layer IV [42]. The transentorhinal zone [43] was not included in this study. Neurons were measured within four layers of the EC. Layer II consists of islands of large modified pyramidal and stellate neurons separated by an acellular gap from layer III. A broad layer III is built up of medium-sized pyramidal neurons separated from layer V by the lamina dissecans, labeled as layer IV. Layer V consists of the large pyramidal neurons, whereas Layer VI is built up of the smallest neurons with a decreasing gradient of neuronal density in the deep portion of this layer [42].

In Ammon’s horn the number of neurons was estimated in the pyramidal layer in sectors CA1–4. The CA1 sector pyramidal neuronal layer extends from the subiculum to the CA2 sector as a thick band of medium size pyramidal neurons. Two features distinguish the CA2 sector: a compact arrangement of large pyramidal neurons and the lowest thickness of this layer. The size of neurons in the CA3 and 4 is similar, but parallel arrangement of neurons in the CA3 sector distinguishes CA3 from randomly dispersed neurons in the hilus, identified also as CA4 sector [42,44]. The mapping of sectors on serial sections was based on the Amaral and Insausti [42] classification, but the CA4 sector has been delinated as a separate subdivision.

The neurons were distinguished from glial cells by using histological features revealed by cresyl violet staining, including neuron size, shape, and spatial orientation typical for specific layers, sectors, and brain nuclei, as well as the pattern of staining of nuclear chromatin, the distinct nucleolus in the majority of examined neuronal populations, and cytoplasm morphology. Small and round nuclei with uniform, intense staining of nuclear chromatin distinguished oligodendrocytes from astrocytes with large, round nuclei with a small amount of dispersed chromatin and an undetectable nucleolus.

### Stereological analysis

Volume evaluation and counting of neurons were performed using a workstation consisting of an Axiophot II (Carl Zeiss, Goettingen, Germany) light microscope with Plan-NEOFLUAR objectives—1.25 × (numerical aperture, N.A., 0.035), 2.5 × (N.A. 0.075), 5 × (N.A. 0.15), 20 × (N.A. 0.5), and 40 × (N.A. 0.75); a specimen stage with a three-axis, computer-controlled stepping motor system (Ludl Electronics; Hawthorne, NY, USA); a CCD color video camera (CX9000, MicroBrightfield Bioscience, Inc., Williston, VT, USA); and stereology software (Stereo Investigator, MicroBrightfield Bioscience).

The parameters and procedures that were applied to estimate the volume of brain regions and the number of neurons are summarized in Table 1. Brain structures and their subdivisions were delineated at a low magnification of 1.25 × (47 × final mag.), 2.5 × (95x), or 5 × (190x) to determine the volume of the region of interest. The number of neurons was estimated by using a 40x lens (final magnification, 1,450x), and the number of Purkinje cells, by using a 20x lens (final magnification, 720x). The numerical density of Purkinje cells (n/mm^3^) was estimated in the cerebellar cortex, including molecular and granule cell layers. An optical fractionator systemic random sampling scheme was applied (Stereo Investigator, MicroBrightfield). Twelve equidistant serial sections were used for neuronal counts in the anatomical subdivisions of the amygdala; 10 in the substantia nigra; 14 in cornu Ammonis sectors CA1–CA4; 13 in the claustrum; 16 in the magnocellular basal complex; 6 in the thalamus, entorhinal cortex, globus pallidus, and nucleus accumbens; 9 in the caudate nucleus and putamen; 8 in the cerebellum and in the inferior olive; and 11 in the dentate nucleus. The bias related to sectioning defects was reduced by application of 5-μm-wide top and bottom guard zones. The results of examination of the cytoarchitectonic subdivisions are presented as the mean number of neurons per mm^3^ (neuronal density) and the total number of neurons per region of interest. The sizes of the grid and of the virtual counting space were adjusted to the size and shape of the examined regions. The number of virtual counting spaces ranged from 62 per subdivision per case in a very small subdivision such as the fifth layer in the entorhinal cortex (coefficient of error; CE, 0.003) to 1953 in the globus pallidus with a low neuronal numerical density (CE, 0.04).

**Table 1:** **Parameters and procedures applied to estimate the volume of brain subdivisions and number of neurons**

**Structures and their subdivisions**	**Number of sections examined (per case)**	**Obj. (Vol.)**	**Obj. (Numb.)**	**Grid size (μm) for fractionator**	**Test area frame × height (μm)**	**Number of counting spaces (per case)**	**Number of neurons counted (per case)**	CE*
Amygdala (four nuclei)	12	1.25x	40x	1000 × 1000	80 × 80 × 10	356	454	0.01
Thalamus	6	1.25x	40x	1000 × 1000	80 × 80 × 10	188	313	0.01
Entorhinal c.								
LII	6	2.5x	40x	400 × 400	60 × 60 × 30	113	164	0.003
LIII	6	2.5x	40x	800 × 800	60 × 60 × 30	118	277	
LV	6	2.5x	40x	600 × 600	60 × 60 × 30	62	161	
LVI	6	2.5x	40x	800 × 800	60 × 60 × 30	68	184	
CA1	14	2.5x	40x	1000 × 1000	60 × 60 × 30	207	543	0.003
CA2	14	2.5x	40x	400 × 400	60 × 60 × 30	121	337	
CA3	14	2.5x	40x	400 × 400	60 × 60 × 30	144	330	
CA4	14	2.5x	40x	600 × 600	100 × 100 × 30	130	429	
Caudate nucleus	9	2.5x	40x	1400 × 1400	60 × 60 × 30	270	751	0.04
Putamen	9	2.5x	40x	1400 × 1400	60 × 60 × 30	309	844	
Globus pallidus	6	2.5x	40x	300 × 300	80 × 80 × 30	1953	505	
Nucleus accumbens	6	2.5x	40x	700 × 700	60 × 60 × 30	202	749	
Magnocellular LGB	4	5x	40x	500 × 500	100 × 100 × 30	96	230	0.002
Parvocellular LGB	4	5x	40x	500 × 500	100 × 100 × 30	134	1,418	0.002
Claustrum	13	1.25x	40x	250 × 250	60 × 60 × 10	274	318	0.01
Substantia nigra	10	2.5x	40x	500 × 500	80 × 80 × 30	366	493	0.002
Magnocellular basal complex (Ch1-Ch4)	16	2.5x	40x	500 × 500	80 × 80 × 30	323	507	0.005
Dentate nucleus	11	2.5x	40x	1000 × 1000	180 × 180 × 30	335	792	0.002
Inferior olive	8	2.5x	40x	1000 × 1000	180 × 180 × 30	213	364	0.002
Cerebellum	8	1.25x	20x	1800 × 1800	180 × 180 × 30	1419	773	0.05

Number of sections examined = mean number of equidistant sections examined per structure/case.

Obj. (Vol.) = Objective lens (1.25x, 2.5x, or 5x) used to provide final magnification for planimetry and volumetry.

Obj. (Numb.) = Objective lens (40x or 20x) used for fractionator to estimate neuronal density and total number of neurons.

*CE = the average predicted Coefficient of Error of the neuronal counts (Scheaffer).

The mean volumes, number of neurons, and neuronal density of structures in autistic and control brains were compared in analyses of covariance (ANCOVAs) and repeated measures analysis of variance in which brain weight and, for neuronal counts and density, log age, post-mortem interval, and days of fixation were entered as covariates. Pearson correlations were computed for age and the total number and density of neurons in the autistic and control cohort.

## Results

### Repeated measures analysis of variance

To test the hypotheses that structures differ in the degree to which autism affects, first, the number of neurons in the structure and, second, the volume of the structure, we conducted repeated measures analyses of variance with structure and autistic status as factors, with the potential confounders of brain weight, post-mortem interval, days of fixation, and log age entered as covariates. The significant interaction of structure with autistic status in each case indicates that autism’s effect upon the number of neurons (F[df = 13] = 2.138, p < .018) and upon structural volume (F[df = 13] = 10.278, p < .001) differs among the structures examined.

### Volume of brain subdivisions

**Figure 3:**
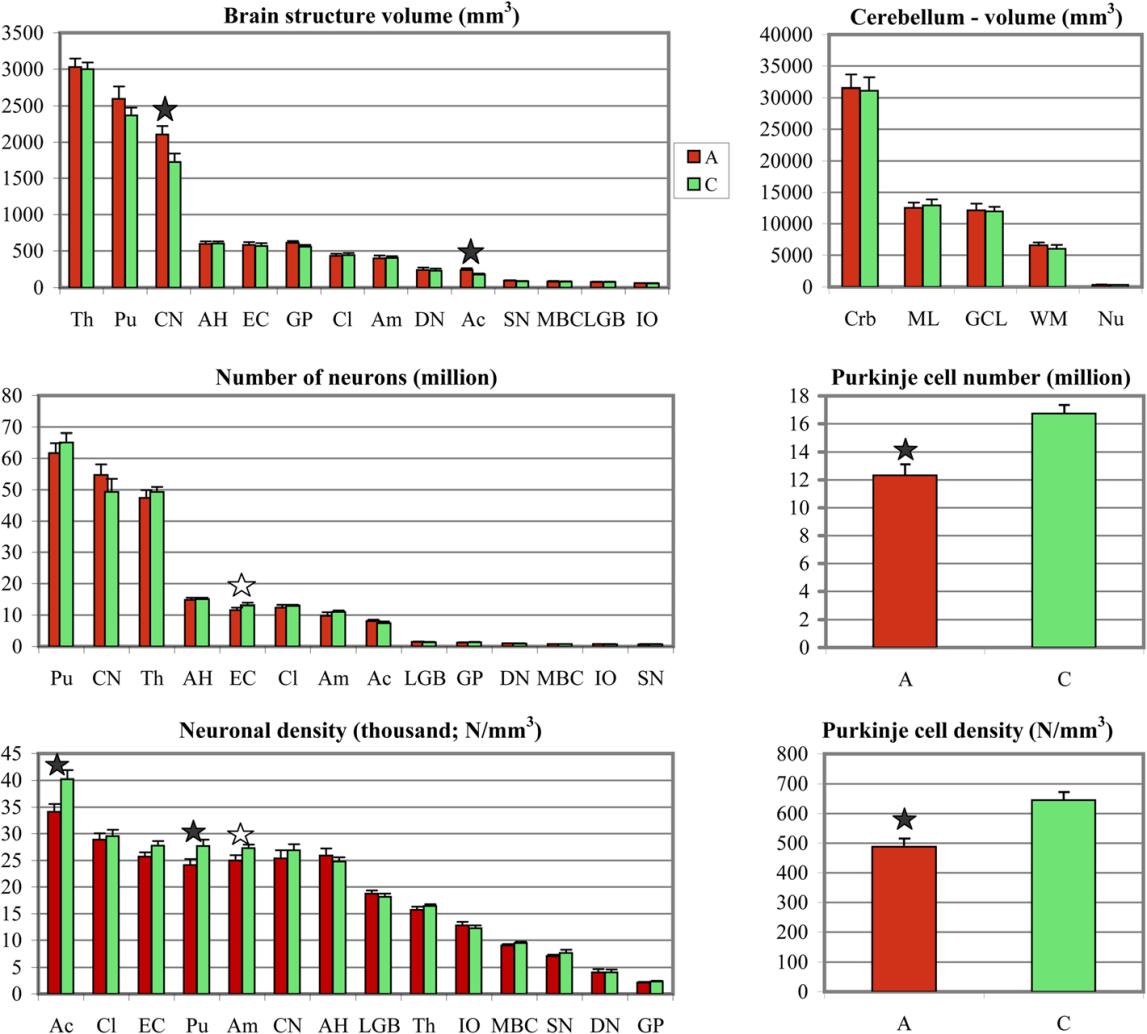
**Brain structure volume, total number of neurons and neuronal density.** Range of the volume of examined regions from the largest (thalamus, putamen, and caudate) to the smallest (inferior olive). The volume of all four striatum subdivisions, including putamen, caudate nucleus, globus pallidus, and nucleus accumbens, is greater in subjects with autism, but the difference in this volume between affected and control subjects is significant only in the caudate and nucleus accumbens (black stars). The total number of neurons was found to be significantly less only in the second layer of the entorhinal cortex (white star) when covariates were not entered in the analysis. Although the total number of neurons does not reveal significant differences, the numerical density is significantly reduced in the nucleus accumbens and putamen of subjects with autism. The significantly lower numerical density of neurons is observed in the lateral nucleus of the amygdala (white star), but not in other amygdala nuclei. Cerebellar volume and the volume of the molecular and granule cell layers, cerebellar white matter, and cerebellar nuclei do not reveal differences between affected and control subjects; however, the total number of Purkinje cells was 25% less (p < 0.03) in the group with autism (12.1 million) than in the control group (16.0 million). The numerical density of Purkinje cells was also significantly less (p < 0.004) in the affected group than in the control group (488/mm^3^ and 645/mm^3^, respectively). Th, Thalamus; Pu, putamen; CN, caudate nucleus; AH, Ammons horn; EC, entorhinal cortex; GP, globus pallidus, Cl, claustrum; Am, amygdala; DN, dentate nucleus; Ac, nucleus accumbens; SN, substantia nigra; MBC, magnocellular basal complex; LGB, lateral geniculate body; IO, inferior olive; Crb, cerebellum; ML, molecular layer; GCL, granule cell layer; WM, white matter; Nu, cerebellar nuclei.

In the control group, the volume of 14 brain structures and their 24 subdivisions ranged from the largest structures, such as the thalamus, putamen, and caudate nucleus (2,999 mm^3^, 2,365 mm^3^, and 1,727 mm^3^, respectively) to the smallest inferior olive (59 mm^3^) (Figure 3). Table 2 summarizes the results of estimation of the volume of brain regions and their cytoarchitectonic subdivisions as the absolute volume calculated using mounted section thickness (StereoInvestigator, Microbrightfield). To compare the results of volume estimates with other reports, the volume was corrected for 47% tissue shrinkage during dehydration for the autism group and 45% shrinkage for the control group. A significant difference between the volumes of the examined structures in the affected and control cases was detected only in the caudate nucleus and nucleus accumbens. In the group with autism, caudate nucleus volume was larger by 22% (2,107 mm^3^ in the autism cohort, and 1,727 mm^3^ in the control cohort; *p* < 0.036). The nucleus accumbens was larger by 34% (244 mm^3^ in the autism cohort, and 182 mm^3^ in the control cohort; *p* < 0.050). However, analysis of alterations in 4- to 8-year-old children did not reveal a significant difference between the affected and control groups. The finding of a significantly larger caudate nucleus and nucleus accumbens only in 9- to 60-year-old individuals with autism suggests that the difference may be a result of changes during adolescence/adulthood.

**Table 2:** Mean volume (±SE) of 14 brain structures and 24 cytoarchitectonic subdivisions in one brain hemisphere of autistic and control subjects*

**Brain structure or cytoarchitectonic subdivision**	**Volume (mm** ^**3**^ **) (Absolute volume calculated using mounted section thickness)**			**Volume (mm** ^**3**^ **) corrected for shrinkage**	
	**Autism**	**Control**	***p*** **<**	**Autism**	**Control**
Thalamus	3,030 ± 116	2,999 ± 92	ns	5,717	5,453
Putamen	2,597 ± 165	2,365 ± 111	ns	4,900	4,300
Caudate nucleus	2,107 ± 114	1,727 ± 114	0.036	3,975	3,140
Cornu Ammonis (CA)	598 ± 33	602 ± 30	ns	1,128	1,096
- CA1	439 ± 22	432 ± 23	ns	828	785
- CA2	32 ± 3	36 ± 2	ns	60	65
- CA3	44 ± 3	45 ± 4	ns	83	82
- CA4	84 ± 7	89 ± 6	ns	158	162
Entorhinal cortex (EC) I-VI	589 ± 39	557 ± 36	ns	1,111	1,012
- EC I	71 ± 4	63 ± 4	ns	134	115
- EC II (Islands)	38 ± 3	42 ± 4	ns	72	76
- EC III	254 ± 17	244 ± 16	ns	479	444
- EC IV	23 ± 3	17 ± 5	ns	43	31
- EC V	60 ± 4	62 ± 5	ns	113	113
- EC VI	143 ± 10	129 ± 9	ns	270	234
Globus pallidus	611 ± 36	561 ± 25	ns	1,153	1,020
- External globus pallidus	412 ± 28	364 ± 29	ns	777	662
- Internal globus pallidus	199 ± 10	197 ± 10	ns	375	358
Claustrum	432 ± 32	442 ± 27	ns	815	804
- Insular claustrum	237 ± 19	241 ± 16	ns	447	438
- Prepiriform claustrum	194 ± 14	201 ± 13	ns	366	365
Amygdala	397 ± 43	405 ± 22	ns	749	736
- Basal nucleus	113 ± 15	116 ± 8	ns	213	211
- Lateral nucleus	189 ± 20	202 ± 13	ns	356	367
- Accessory basal nucleus	82 ± 10	76 ± 6	ns	155	138
- Central nucleus	13 ± 1	11 ± 1	ns	25	20
Nucleus accumbens	244 ± 23	182 ± 13	0.050	460	331
Substantia nigra	98 ± 6	88 ± 4	ns	185	160
Magnocellular basal complex	84 ± 8	81 ± 5	ns	158	147
Lateral geniculate body	80 ± 5	77 ± 4	ns	151	140
Inferior olive	62 ± 2	59 ± 4	ns	117	107
Cerebellum	31,779 ± 1,304	31,133 ± 1,397	ns	59,959	56,605
- Cerebellar cortex (calculated)	24,715	24,811	ns	46,632	45,111
- Molecular layer	12,623 ± 443	12,885 ± 758	ns	23,816	23,427
- Granule cell layer	12,092 ± 611	11,926 ± 474	ns	22,815	21,684
- Cerebellar white matter	6,700 ± 380	6,005 ± 323	ns	12,641	10,918
- Cerebellar nuclei	364 ± 34	317 ± 21	ns	686	576
- Dentate nucleus	244 ± 14	232 ± 13	ns	511	422

*Statistical significance controlled for brain weight and brain weight decrease during dehydration (%) entered as covariate. ns = not significant.

Volume (mm^3^) corrected for shrinkage: autism, 47%; control, 45%.

The mean volume of the examined cerebellar hemisphere was almost identical in the affected and control groups: 31,779 mm^3^ and 31,133 mm^3^, respectively. The mean volume of the molecular and granule cell layers (12,623 mm^3^ and 12,092 mm^3^, respectively), and the percentage of the cerebellar cortex occupied by these two layers (51% and 49%, respectively) in the group with autism did not reveal a significant difference in comparison to the control group (12,885 mm^3^ and 11,926 mm^3^, respectively; with the volume ratio 52% and 48%, respectively). The mean volume of white matter was almost the same in the affected and control cohorts (6,700 mm^3^ and 6,005 mm^3^, respectively), as was the percentage of the cerebellar volume occupied by white matter (21% and 19%, respectively). Also, the volume of cerebellar nuclei (364 mm^3^ and 317 mm^3^, respectively), including the largest dentate nucleus (244 mm^3^ and 232 mm^3^, respectively), did not reveal a significant difference between individuals with autism and control subjects.

### Number of neurons

Application of the fractionator method to determine the total number of neurons in 15 brain structures and their 16 cytoarchitectonic subdivisions in control subjects (Table 3) revealed 49 million to 65 million neurons in the three largest structures, including the thalamus, caudate nucleus, and putamen, and 7 million to 15 million in medium-size structures such as the nucleus accumbens, amygdala, entorhinal cortex, claustrum, and Ammons horn. In the smallest examined brain structures, the number of neurons ranged from 0.7 million in the substantia nigra to 1.4 million in the lateral geniculate body. Examination of 31 brain regions and cytoarchitectonic subdivisions revealed a significantly lower total number of neurons only in the second layer of the entorhinal cortex (813,252 in subjects with autism and 1,023,270 in control subjects; *p* < 0.013 not corrected for log of age, brain weight, postmortem interval, and days of fixation). In two affected cases, neuronal deficit in the entorhinal cortex was detected without the support of morphometric methods, as the paucity of focal stellate neurons in disorganized islands that were smaller than normal. However, a reduction of the mean total number of neurons to 788,879 and the shift of significance to *p* < 0.007 after removal of these two cases from the analysis indicates that these focal developmental defects contribute less to the decrease of the mean number of stellate neurons than do diffuse deficits not detectable without morphometric support.

**Table 3:** Total number of neurons per region and neuronal numerical density in 15 structures and 16 cytoarchitectonic subdivisions in examined brain hemisphere of individuals with autism and control subjects*

**Structure/cytoarchitectonic subdivision**	**Total number of neurons per region**			**Neuronal numerical density (N/mm** ^**3**^ **)**		
	**Autism**	**Control**	***p*** **<**	**Autism**	**Control**	***p*** **<**
Putamen	61,675,039	65,111,021	ns	23,749	27,531	0.041
Caudate nucleus	54,693,459	49,292,390	ns	25,958	28,542	ns
Thalamus	47,403,083	49,291,830	ns	15,645	16,436	ns
Cornu Ammonis (CA)	14,812,043	15,047,614	ns	24,769	24,955	ns
- CA1	11,575,341	11,533,806	ns	26,368	26,699	ns
- CA2	1,051,901	1,152,120	ns	32,872	32,003	ns
- CA3	1,054,780	1,124,862	ns	23,972	24,997	ns
- CA4	1,130,021	1,236,826	ns	13,453	13,897	ns
Claustrum	12,351,088	12,917,584	ns	28,590	29,225	ns
- Insular claustrum	6,576,940	6,729,652	ns	27,751	27,924	ns
- Prepiriform claustrum	5,774,148	6,187,932	ns	29,764	30,786	ns
Entorhinal cortex (EC)	11,878,538	12,915,740	ns	23,997	27,077	ns
- EC Islands	813,252	1,023,270	ns^†^ (0.013)	21,401	24,364	ns
- EC III	5,553,881	6,071,044	ns	21,866	24,881	0.021
- EC V	1,871,129	2,008,421	ns	31,185	32,394	ns
- EC VI	3,640,276	3,813,005	ns	25,456	29,558	ns
Amygdala	9,819,741	11,138,599	ns	24,735	27,503	ns
- Basal nucleus	2,821,429	3,154,350	ns	24,968	27,193	ns
- Lateral nucleus	4,609,751	5,544,098	ns	24,390	27,446	0.017
- Accesory basal nucleus	2,046,439	2,109,385	ns	24,957	27,755	ns
- Central nucleus	342,122	330,766	ns	26,317	30,070	ns
Nucleus accumbens	8,078,093	7,285,654	ns	33,107	40,031	0.018
Lateral geniculate body	1,493,426	1,392,966	ns	18,668	18,090	ns
Globus pallidus	1,277,936	1,312,262	ns	2,092	2,339	ns
- External globus pallidus	899,307	904,365	ns	2,183	2,485	ns
- Internal globus pallidus	378,629	407,897	ns	1,903	2,071	ns
Dentate nucleus	970,440	918,809	ns	3,977	3,960	ns
Magnocellular basal complex	768,415	790,893	ns	9,148	9,764	ns
Inferior olive	793,002	716,895	ns	12,790	12,151	ns
Substantia nigra	744,247	707,228	ns	7,594	8,037	ns
Cerebellum (Purkinje cells)	12,060,920	16,003,095	0.0001	488	645	0.0024

*Controlled for log age, brain weight, post-mortem interval, days of fixation, and percentage of brain weight loss during dehydration entered as covariates.

^**†**^The total number of neuronal deficit (by 20%; *p* < 0.013) in stellate neuron islands in the entorhinal cortex was not significant only when log age, brain weight, post-mortem interval, and days of fixation were entered as covariates.

**Figure 4:**
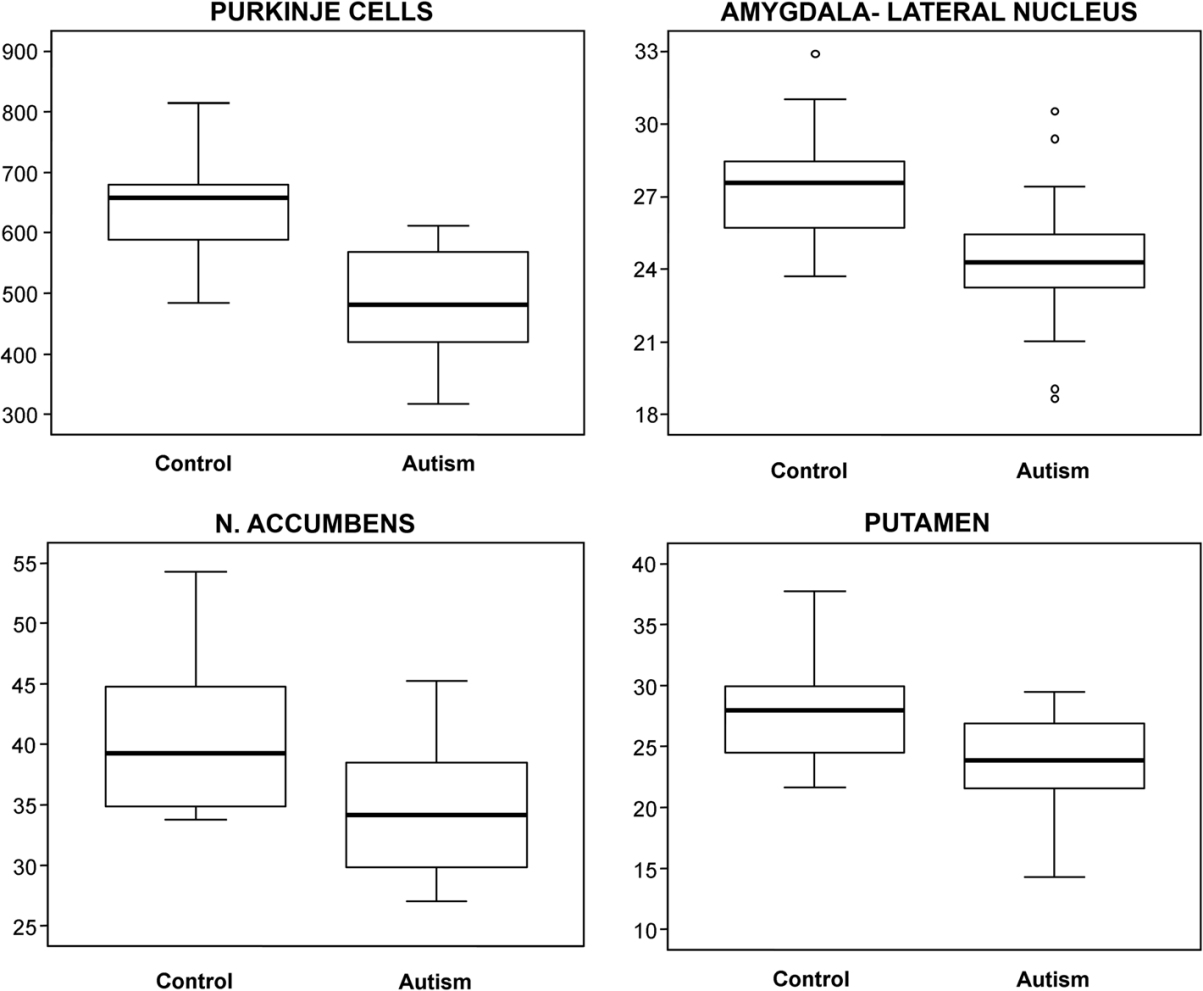
**Numerical density of Purkinje cells, and neurons in the lateral nucleus in the amygdala, nucleus accumbens and in the putamen.** Box plots demonstrate the considerable differences between the median numerical density (thick line within the box) of Purkinje cells (n/mm^3^), and between the number of neurons in the lateral nucleus in the amygdala, nucleus accumbens and putamen (thousands/mm^3^) in the group with autism and control groups. In the majority of individuals diagnosed with autism, the numerical density of neurons is less than the median numerical density in the control group. The upper and lower boundaries of each box represent the 75th and 25th percentiles of the data, respectively; the depth of the box thus represents the interquartile range (IQR). The “whisker” above the box is the maximum value unless any data point lies more than 1.5 times the IQR above the 75th percentile, in which case the maximum value is the 75th percentile plus 1.5 times the IQR, and the points outside it (outliers) are indicated by circles. The lower whisker and outliers are computed analogously.

The numerical density of neurons was brain region–specific and varied in the control group from 2,339/mm^3^ in the globus pallidus to 40,031/mm^3^ in the nucleus accumbens. In the group with autism, a significantly lower numerical density of neurons was detected in three brain regions: the lateral nucleus in the amygdala (24,390/mm^3^ in the affected group and 27,446/mm^3^ in the control group; *p* < 0.017), putamen (23,749/mm^3^ in the affected group and 27,531 mm^3^ in the control group; *p* < 0.041), and nucleus accumbens (33,107/mm^3^ in the affected group and 40,031/mm^3^ in the control group; *p* < 0.018). Box plots demonstrate that in the majority of subjects with autism the numerical density of Purkinje cells, neurons in the lateral nucleus in the amygdala, nucleus accumbens and in the putamen is less than the median numerical density in the control group (Figure 4).

The volume of cerebellum and cerebellar subdivisions as well as the number of neurons was estimated after exclusion of records for brain samples of two subjects with autism: one with focal defects of cresyl violet staining related to poor fixation, and the second because of focal dysplasia. A 60-year-old subject with autism and clinically diagnosed disturbed movement coordination and postmortem-detected severe hypoplasia of cerebellar lobes I-IV [36] was excluded from statistical analysis to eliminate the impact of one case of focal developmental defect on estimates of developmental alterations in an examined group of subjects with autism. In this cerebellum, the numerical density of Purkinje cells was 2.3 times less (274/mm^3^) in the hypoplastic area than in the control cerebellar cortex. The total number of Purkinje cells was 42% less in this case (7.1 million) than the mean total number in the examined hemisphere of other subjects with autism and 65% less than in the control group.

The average total number of Purkinje cells within the examined cerebellar hemispheres was significantly less (*p* < 0.0001) in the group with autism (12.1 million) than in the control cohort (16. million) (25% deficit). The minimal number of Purkinje cells in the examined hemisphere in the control group was 11.4 million, but in five of 12 examined subjects in the affected group (42%), the number was less than in this control subject. The total number of Purkinje cells was less in all individuals with autism than the average number in the control group. The numerical density of Purkinje cells was also significantly less (*p* < 0.0024) in affected subjects (488/mm^3^) than in control subjects (645/mm^3^) (−24.4%).

The absence of a significant correlation between age and the total number or numerical density of neurons (N/mm^3^) in the cohort with autism suggests that the detected neuronal deficits do not decrease greatly, if at all, from 4 to 60 years of age among subjects diagnosed with autism. However, examination of only three subjects with autism 52 to 60 years of age and of three control subjects 51–64 years of age is not favorable for detection of aging-associated neuronal loss.

## Discussion

Estimates of the volume of 38 brain subdivisions, neuronal number per cubic millimeter, and neuronal number per specific cytoarchitectonic subdivision revealed that significant alterations in the group of subjects with autism are limited to the caudate nucleus and nucleus accumbens in the striatum, the lateral nucleus in the amygdala, and Purkinje cells in the cerebellum. The pattern of developmental alterations of the brain structures’ volume and number of neurons limited to these few brain regions is consistent with several morphometric and MRI studies, but is in striking contrast to the abnormal trajectories of neuronal soma volume growth detected in 14 of 16 examined brain structures of 4- to 8-year-old children with autism. The number of regions with a significant deficit of neuronal soma volume decreases to three in autistic teenagers/young adults and has been observed in only four regions in 36- to 60-year-old autistic individuals [35]. This disparity between the limited alterations of brain structures’ volume and number of neurons but the common neuronal volume deficit in children and an increase of perikaryon volume in autistic teenagers and adults suggests a separation of mechanisms controlling developmental changes of the number of neurons and of brain structure volume from mechanisms that control brain region–specific trajectories of neuron growth. These two studies suggest that some neuronal populations—Purkinje cells, neurons in the caudate nucleus and accumbens, and in the lateral nucleus of the amygdala—are affected by both mechanisms.

### Cerebellum

Studies of the cerebellum of individuals diagnosed with autism provide inconsistent results, including smaller cerebellar hemispheres [45,46] and smaller vermis [47-49] as well as increased cerebellar volume or volume proportional to brain size [50]. The study by Fatemi et al. [25] of the cerebellum of five males with autism with a mean age of 25.2 years and of five age-matched controls revealed a 24% decrease in mean Purkinje cell size but no differences in Purkinje cell densities. The study of the cerebellum of a 16-year-old female diagnosed with autism and severe intellectual deficit showed no alterations in the cerebellum [24]. Several reports suggested associations between the size of the vermis and attention deficits, enhanced stereotypic behavior, and reduced exploration in autism [51-53].

Our review of the literature indicates that the current study is the first to report on the total number of Purkinje cells and cerebellar global volumetry in autism. The study revealed a significantly lower total number of Purkinje cells in the cerebellum in the group of subjects who were diagnosed with autism (12.1 million per examined hemisphere, and 24.2 million calculated for entire cerebellum) than in control subjects (16.0 and 32.0 million, respectively). The total number of Purkinje cells in the entire cerebellum of control subjects in our cohort 4–64 years of age is comparable to estimates reported by other authors, with some modifications due to age and methods: 28.5 million in 10 control males, 28–84 years of age [54]; 28.6 million in 19 control subjects, 19–84 years of age [55], 29.8 million in 10 controls, 21–65 years of age [56]; and from 19.7 to 27.0 million in four controls, 41–84 years old [57].

Autism is associated with significant inter-individual differences in the number of Purkinje cells, but a smaller number of Purkinje cells was observed in the majority of 29 individuals diagnosed with autism (reviewed by Palmen et al., [58]). Recently, eight subjects with autism and seven control subjects from our cohort of 14 autistic and 14 control subjects were examined by Skefos et al. [59] to detect links between structural alteration of four cerebellar subdivisions and functional abnormalities typical for autism. The authors examined the crus I and II in the posterior lobe reciprocally connected with prefrontal cortical networks that modulate social behavior, and the hemispheric portion of lobules IV-VI known as a primary sensorimotor processing area. The lower Purkinje cell density in these regions supports the hypothesis that abnormal Purkinje cell density may contribute to selected clinical features of the autism phenotype. The reduced number of Purkinje cells and dysplastic changes in lobule X may have a direct contribution to alterations of eye movement in subjects with autism [60]. Some studies suggest that a regional decrease in the number of Purkinje cells may be the result of prenatal loss of Purkinje cells [27]. Some inter-individual differences in the number of Purkinje cells reported in the past might be related to treatment of epilepsy-associated loss of Purkinje cells [61-64] and/or pre-mortem changes related to the mechanism of death. Signs of structural developmental alterations coincide with a 40% decrease in the expression of glutamic acid decarboxylase 67 (GAD67) mRNA in autistic subjects and an increased GABAergic feed-forward inhibition to Purkinje cells by basket cells. These data suggest a disruption in the timing of Purkinje cell firing and altered inhibition of the cerebellar nuclei, which could directly affect cerebello-cortical output, leading to changes in motor behavior and cognition [65].

In the examined cohort, the difference between volumes of the cerebellar hemisphere in the group with autism and control groups (31,779 mm^3^ and 31,133 mm^3^, respectively) was not significant. This volume reflects a 45% reduction of brain weight during dehydration in ethyl alcohol of the brains of control subjects and a 47% reduction in individuals diagnosed with autism. The volume calculated for both cerebellar hemispheres of control subjects and corrected for 45% shrinkage is equal to 113.2 mm^3^ and is comparable to 124.8 cm^3^ in the study by Andersen et al. [55] of 19 control males from 19 to 84 years of age.

This study found a significant—25%—deficit in the number of Purkinje cells in the absence of significant differences between affected and control cohorts in the volume of the molecular and granule cell layer, cerebellar white matter, or total volume of cerebellar nuclei. Previous study revealed severe Purkinje cell soma volume deficits in the range of 31% in 4- to 8-year-olds and of 23% in 29- to 60-year-olds [35]. These findings, and the absence of abnormalities in the number and distribution of GABAergic basket and stellate interneurons in the cerebellar molecular layer reported by Whitney et al. [27], suggest that Purkinje cells are the major target of the genetic and/or epigenetic factors that define the contribution of Purkinje cells’ pathology to the autistic phenotype.

### Striatum

In our control cohort the volume of the caudate nucleus was estimated as 1,727 mm^3^, versus 2,212 mm^3^ in Kreczmanski et al. [66] study, and 2,043 mm^3^ in Bogerts study [67] (with Kreczmanski’s et al. correction for shrinkage). The volume of the putamen was 2,365 mm^3^ in our material, 2,713 mm^3^ in Kreczmanski et al. study [66], and 2,979 mm^3^ in Bogerts study [67] (with Kreczmanski's correction for shrinkage). The volume of the nucleus accumbens was estimated as 182 mm^3^ in this study, 204 mm^3^ in Kreczmanski et al.[66], 147 mm^3^ in Lauer et al. [68] (with Kreczmanski’s shrinkage factor), and 138 mm^3^ in Pakkenberg study based on examination of 4-μm thick paraffin sections [69].

The volume of the caudate nucleus was significantly greater in the group with autism than in the control group (2,107 mm^3^ and 1,727 mm^3^, respectively; p < 0.036). Several MRI studies of patients with autism have revealed increased volumes of the basal ganglia [20,70-72] and a correlation between caudate nucleus volumes and repetitive-behavior scores [70,71]. A direct relationship between repetitive behaviors and right caudate nucleus volume was reported in an cohort of 17–55 years of age patients diagnosed with autism [71], but not in a cohort including children [73]. Brain size increases to the age of 5 years, whereas the volume of the caudate grows until late childhood [18,74] and decreases during adulthood [73,75]. Regional reduction of the caudate nucleus head volume by up to 50% and the presence of foci of tissue loss, detected by repeated scanning with high-resolution MRI and use of continuum mechanical tensor maps, were interpreted as evidence of the pruning process at 9–13 years of age in control subjects [74]. The lack of differences on postmortem evaluation of caudate nucleus volume between 4- to 8-year-old autistic and age-matched control subjects, but the significantly larger caudate nucleus and nucleus accumbens and insignificantly higher (by 11%) total number of neurons in these structures in 9- to 60-year-old subjects diagnosed with autism in our cohort may indicate timely pruning in the control group, but regionally defective pruning in affected subjects, as postulated by Voelbel et al. [76].

A subcomponent of changes in the striatum is the significant deficit in the volume of neuronal soma in the nucleus accumbens (34%) and caudate nucleus (22%) in 4- to 8-year-old subjects with autism, but not in affected adolescents or adults [35]. These data suggest the deregulation of mechanisms that control the number and volume of neurons and the total volume of the caudate nucleus and nucleus accumbens. Correlations between MRI-detected alterations within the striatum and the severity of clinical changes [70,71] imply that these cellular alterations may contribute to stereotyped and ritualistic behaviors in autism. The nucleus accumbens, which is considered a mixed structure with elements of the striatum and the amygdala [77], processes information about reward value and availability and is involved in reward-related behaviors [78]. Projections of nucleus accumbens neurons to the ventral pallidum translate reward information into motivated action [79]. A significantly larger nucleus accumbens (by 34%, *p* < 0.050) appears to be another deregulated component of striatal circuits that may enforce repetitive behaviors in autism.

The striatum alterations observed in idiopathic autism resemble some structural and functional changes observed in fragile X syndrome (FXS). Autism is diagnosed in 25–30% of individuals diagnosed with FXS [80-84]. Hatton et al. [82] suggested that lower levels of fragile X mental retardation protein (FMRP) expression contribute to autistic behavior and intellectual deficits in children with FXS. Gothelf et al. [85] revealed a correlation between lower levels of FMRP, behavioral alterations, and alterations of the size of some brain subdivisions, including increased size of the caudate nucleus but decreased size of the amygdala, superior temporal gyrus, and posterior cerebellar vermis in FXS. A 75% reduction of FMRP expression in vermis and a 50% reduction in the superior frontal cortex (BA9) in adults with idiopathic autism may contribute to both structural abnormalities and the autistic phenotype [86].

### Amygdala

Neuropathological studies [87] and results of structural [10,88] and functional [89] neuroimaging indicate that the amygdala is affected in autism. The study by Schumann and Amaral [31] of the amygdala in nine brains of individuals with autism 10–44 years of age and 10 brains of age-matched controls did not reveal a difference in the number of neurons in the basal, accessory basal, and central nucleus. However, the authors reported 13% fewer neurons in the lateral nucleus (3.47 million in the autistic group and 4.00 million in the control group; *p* < 0.05) and 12% fewer neurons in the entire amygdala (10.74 million in the affected group and 12.21 million in the control group; *p* < 0.05). The significantly (*p* < 0.017) reduced numerical density of neurons in the lateral nucleus in the amygdala of subjects with autism in our cohort of 14 subjects 4–60 years of age (24,390/mm^3^ in the affected group and 27,446/mm^3^ in the control group) and the absence of significant changes in other amygdala subdivisions in these subjects confirm the selective developmental abnormality of the lateral nucleus. In our study, the total number of neurons in the lateral nucleus in subjects with autism was also less (by 17%) (4,609,751) than in the control group (5,544,098), but after control for five covariates, the difference was found to be not significant. Differences in tissue preservation, the age of examined subjects, and selection of only epilepsy-free males in the study by Schumann and Amaral [31] may contribute to some differences in the number of neurons reported, but the general pattern of alterations detected in both studies is similar.

Reduced neuronal density in the absence of a change in the total number of neurons in the amygdala may suggest the involvement of non-neuronal factors, including glia. Ahlsen et al. [90] reported increased levels of GFAP, a marker of astroglial activation, in the CSF in subjects with autism. There are no data about astrocytosis within the amygdala, but Laurence and Fatemi [91] demonstrated by Western blots an increased level of GFAP in superior frontal cortex (BA9; by 45%), parietal cortex (BA40; by 75%) and in the cerebellum (by 49%) in subjects with autism. Vargas et al. [92] reported signs of selective activation of astroglia and microglia in several brain regions of subjects with autism. A new concept of tripartite synapses with astrocytes controlling synapses formation and function [93] and signs of astrocytosis may suggest a glial contribution to structural and functional changes in the brains of subjects diagnosed with autism.

Individuals with autism display deficits of social behavior, including abnormalities in social reciprocity, difficulties in the use of eye contact, and deficits in social motivation [1]. The theory of mind proposes that the amygdala is one of several brain regions that are parts of the social brain and are necessarily abnormal in autism [89]. Moreover, the amygdala controls behavioral responses such as the fear and anxiety observed in 84% of children with autism [94,95]. The alterations in the amygdala detected in morphometric studies and in MRI-based volumetry suggest that developmental abnormalities of the amygdala may contribute to the autistic phenotype. Some differences of the estimated total number of neurons in the lateral nucleus in the amygdala in the control group in Schumann and Amaral [4] study (4.0 million), Kreczmanski et al. [66] (4.43 million) and 5.54 million in our study reflect mainly technical differences and in part interindividual differences.

### Neocortex and archicortex

Stoner et al. study [96] revealed patches of disorganization in the prefrontal and temporal cortices in a majority of children diagnosed with autism. This focal disruption of cortical architecture suggests dysregulation of layer formation and layer-specific neuronal differentiation at prenatal developmental stages. Van Kooten et al. [32] showed significant reductions in neuron densities in layer III, total number of neurons in layers III, V, and VI in the fusiform gyrus, but none of these alterations were found in the primary visual cortex or the whole cerebral cortex. This study provides insight about the cellular basis of abnormalities in face perception in autism. Several other reports indicated the absence of alterations in the number of neurons in the cerebral cortex [97]; in the superior frontal cortex [98]; in the BA44 and BA45 [28]; and in the frontoinsular cortex [23]. A study of von Economo neurons revealed no significant difference in the mean number of these neurons in the frontoinsular cortex [26] and in the anterior cingulate cortex (BA24) [33]. A study of the frontoinsular cortex in four subjects with autism, (4–11 years of age) and three control (4–14 years of age) subjects revealed an increase in the ratio between von Economo neurons and pyramidal neurons, but the difference between the number of von Economo neurons in subjects with autism and control subjects fell short of statistical significance, and the total estimated number of pyramidal neurons did not reveal a significant difference [23].

Our study of the archicortex of subjects with autism revealed focal microdysgenesis with selective deficits of stellate neurons in irregular smaller islands in the second layer of the entorhinal cortex in 23- and 60-year-old affected subjects [36]. The reduced total number of neurons in the islands and in the third layer of the entorhinal cortex suggests developmental abnormalities within the perforant pathway projecting to the dentate gyrus and Ammons horn. These alterations may affect memorization and emotional behavior [99]. Similar deficits of stellate neurons in islands and a thicker molecular layer in the entorhinal cortex of subjects diagnosed with schizophrenia were described as evidence of defects of migration of neurons to their target destination during development [100,101].

### Developmental deficit and loss of neurons in autism

For much of the history of postmortem studies of ASD pathology, brain abnormalities have been viewed as static [102], but the regression observed in 15–62% of autism cases has been linked to neurodegeneration and neuronal loss [103]. However, morphological markers of neurodegeneration have been reported only sporadically, including cytoplasmic homogenous inclusions in Purkinje cells in affected child [98], Purkinje and granule cells’ mineralization [87], and neurofibrillary degeneration in a 24-year-old autistic female with self-injurious behavior including head-banging [104].

The Fatemi et al. [105] review demonstrates agreement on a Purkinje cells deficit in the cerebellum of individuals diagnosed with autism. The lower number of Purkinje cells in subjects diagnosed with autism is considered mainly the result of the loss of these cells before 30 weeks’ gestation [87], but postnatal Purkinje cell death accompanied by astrocytosis has been also reported [98]. In some cases, loss of Purkinje cells might be the result of chronic treatment with anti-epileptogenic drugs such as phenytoin [61,62]. The lack of correlation between the number of Purkinje cells and age in the examined cohort of autistic subjects 4–60 years of age suggests that the 25% deficit of Purkinje cells is mainly a developmental fetal defect, as postulated by Kemper and Bauman [87], but the combination of prenatal deficit and postnatal loss cannot be excluded.

Comparison of the total number of neurons in the Ammons horn in this control group and control groups in other reports reveals differences related to the methods of tissue preservation and morphometric evaluation, and the age of examined cohorts. Our study revealed that the total numbers of neurons in the CA1, CA2/3, and CA4 sectors of control subjects 4–64 years of age were 11.5, 2.1, and 1.1 million, respectively. The numbers of neurons in the CA1, CA2/3, and CA4 sectors in control subjects 21–56 years of age were determined to be 14.7, 2.7, and 2.12 million, respectively [106]; for 47– 85 years of age, 16.0, 2.7, and 2.0 million, respectively [107]; and for 72–96 years of age, 6.2, 1.7, and 0.7 million, respectively [108]. Similar, age-associated differences are observed in the substantia nigra, with the total number of neurons of 707,000 in our control group, and of 550,000 neurons in 71- to 87-year-old control subjects in the Pakkenberg et al. study [109].

Several studies have revealed neuronal pathology affecting cytoplasmic organelles and possibly sustaining functional abnormalities in autism, but there is no direct evidence that they cause significant neuronal death. An abnormal electron transfer chain in neuronal mitochondria may alter energy generation, metabolism, and cell structure and function; however, these changes prevail only in children, not in adults [110,111]. An imbalance between mitochondria biogenesis and degradation increases the mass of compromised mitochondria in neurons in the temporal cortex, but this pathology was also demonstrated only in young individuals diagnosed with ASD [112]. Currently, there is no evidence that the elevated oxidative stress detected as an increase of 3-nitrotyrosine in the cortex, cerebellum, and pons of subjects with autism [113] and the two-fold increase of DNA oxidation in the frontal and temporal cortex and in the cerebellum [114] cause neuronal loss. In idiopathic autism and autism associated with dup15, a self-enhancing cascade of pathological changes alters amyloid precursor protein (APP) metabolism, and increases cytoplasmic accumulation of amino-terminally truncated amyloid-β, which is the source of reactive oxygen species and which increases the formation of lipid peroxidation products. These changes enhance amyloid-β deposition and sustain the cascade of changes contributing to metabolic and functional impairments in neurons [115,116]. These alterations may contribute to activation of astrocytes and microglial cells, enhanced astrocytosis, and astrocytes’ death [115]; however, the potential neuronal loss associated with this pathology appears to be hidden within a range of interindividual differences and was not detected as an age-associated correlate in the examined cohort.

## Conclusions

The alterations in regional volume and neuron number in only a few brain structures in the autism cohort we studied here contrast with the brain region–specific developmental deficits in the volumes of neuronal soma detected in almost all examined brain regions as shown in our previous report [35]. This disparity may reflect a separation of mechanisms controlling developmental changes in neuronal number and brain structures’ volume from mechanisms controlling brain region–specific trajectories of neuronal growth. However, the severe deficit of Purkinje cell, and significant alterations of the volume of striatum and amygdala subdivisions and their neuronal number, combined with prominent alterations of trajectories of neuronal growth, suggest that developmental abnormalities of these brain structures are products of mechanisms controlling both structures’ size and neuronal number and volume. This duplication of mechanisms controlling the different types of developmental abnormalities in a few brain regions may amplify their contribution to the repetitive behaviors and social and communication deficits observed in autism.

This study increases the coherence of comparative stereological characteristics by the assessment of 38 brain structures and cytoarchitectonic subdivisions in a relatively large cohort of subjects diagnosed with autism using one standard of tissue preservation, embedding, sectioning, and staining and unbiased stereological estimates. The consistency of the characteristics of this cohort is enhanced by our previous analysis of qualitative developmental abnormalities [36], the study of trajectories of neuronal soma changes throughout the lifespan [35], and the application of a major portion of this material for unbiased stereological studies of developmental abnormalities in the cerebral cortex [23,28,32], cerebellum [59], and brainstem [34].

## Consent

This postmortem study has been performed using anonymous coded brain tissue samples. Selected clinical records were extracted from the anonymous coded Autism Tissue Program - Autism Speaks database by authorized project PI.
